# Death-associated protein kinase 1: a double-edged sword in health and disease

**DOI:** 10.3389/fimmu.2025.1593394

**Published:** 2025-08-21

**Authors:** Xiuli Zhang, Yehuda G. Assaraf, Yao Lin

**Affiliations:** ^1^ The Affiliated People’s Hospital of Fujian University of Traditional Chinese Medicine, College of Integrative Medicine, Fujian-Macao Science and Technology Cooperation Base of Traditional Chinese Medicine-Oriented Chronic Disease Prevention and Treatment, Fujian-Hong Kong-Macau-Taiwan Collaborative Laboratory for the Inheritance and Innovation of Traditional Chinese Medicine, Fujian University of Traditional Chinese Medicine, Fuzhou, Fujian, China; ^2^ The Fred Wyszkowski Cancer Research Laboratory, Faculty of Biology, Technion-Israel Institute of Technology, Haifa, Israel

**Keywords:** DAPK1, biological processes, small molecule, guardians of inflammation, health

## Abstract

Death-associated protein kinase 1 (DAPK1) is a Ca^2+^/calmodulin-regulated serine/threonine kinase that orchestrates a wide array of cellular activities. It is intricately regulated through multiple mechanisms, including intramolecular signaling and interactions with other proteins, such as kinases and phosphatases. DAPK1 plays a pivotal role in regulating various biological processes, including apoptosis and autophagy, and is implicated in pathogenesis of several disorders, such as cancer, stroke and brain damage, neurodegenerative and within their kinase domains. In 2014, a collection of reviews was cardiovascular diseases, wound healing, kidney injury, and tuberous sclerosis complex. In light of its biological significance, several small molecule modulators of DAPK1 have been developed for therapeutic purposes and as probe compounds to enhance the mechanistic understanding of DAPK1-mediated biological functions. However, the repertoire of available small molecules remains limited, underscoring the need for further research to discover novel strategies for the activation or inhibition of DAPK1. From this perspective, we primarily discuss the structure, biological function, and role of DAPK1 in health and disease, as well as the recently identified small molecule inhibitors and activators. This analysis offers valuable insights for advancing research in the DAPK1 field.

## 1 Introduction

**Figure 1:**
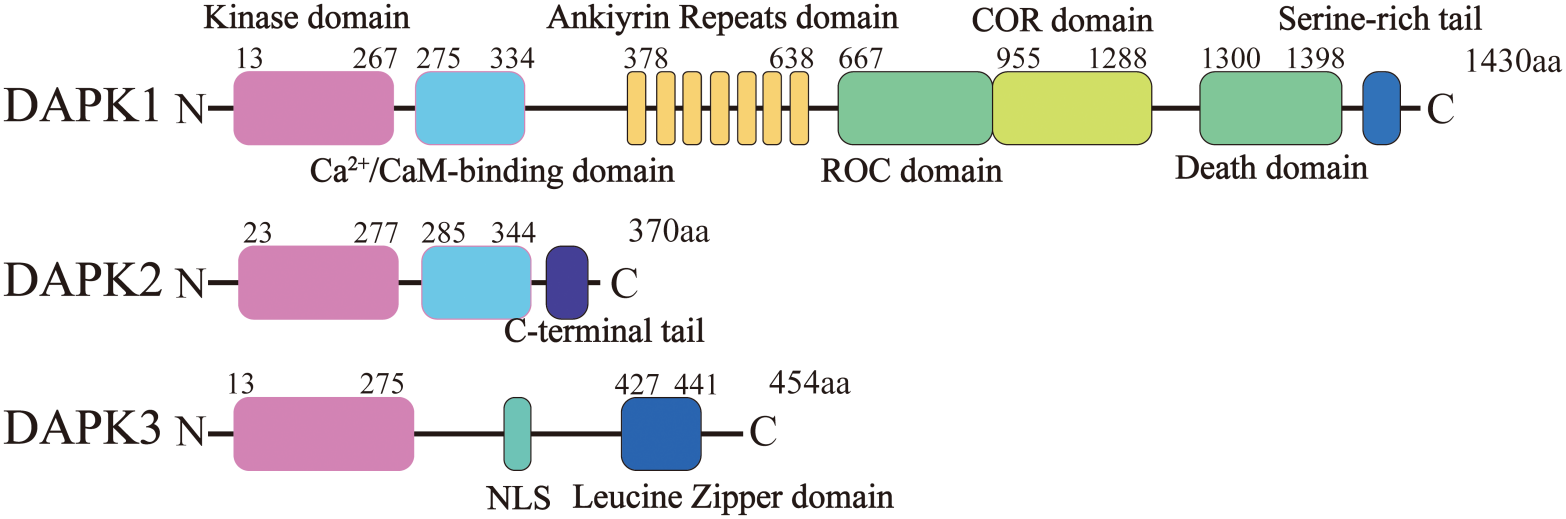
Structural domains of DAPK1 and its homologs DAPK2 and DAPK3. This figure illustrates the domain organization of DAPK1, DAPK2 (DRP-1), and DAPK3 (ZIPK). DAPK1 consists of 1,430 amino acids and contains several distinct domains from the N-terminus to the C-terminus: a kinase domain, a calmodulin (CaM) regulatory domain, a region of ankyrin repeats (comprising eight repeats), a Ras of complex (ROC-COR) domain, a death domain (DD), and a serine-rich tail. DAPK2 and DAPK3 share significant amino acid homology with DAPK1 within their kinase domains (over 80%).

In 1995, death-associated protein kinase 1 (DAPK1), also referred to as DAPK, was initially identified by Adi Kimchi and her colleagues during their quest for genes essential for interferon (IFN-γ)-induced cell death (1). This discovery was made using an antisense library and HeLa cells (1). Subsequently, two additional kinases were identified that exhibited > 80% amino acid homology with DAPK1 within their kinase domains: DAPK2, also known as DAPK-related protein 1 (DRP-1), and DAPK3, also known as zipper-interaction protein kinase (ZIPK) (2, 3) (**Figure 1**). The DAPK1–3 family belongs to a kinase superfamily due to the significant conservation observed within their kinase domains. In 2014, a collection of reviews was published in the journal *Apoptosis* to commemorate the 20th anniversary of the discovery of DAPK1 (4–16). These reviews provided an in-depth discussion of the advancements made in the DAPK1 field from 1995 to 2013. The current review aims to focus on the recent developments concerning DAPK1 over the past decade, emphasizing the functional roles of human DAPK1 (unless otherwise stated) in health and disease.

The human DAPK1 gene maps to chromosome 9 at the 9q34.1 locus and encompasses 26 exons (17). The complete DAPK1 mRNA sequence spans 4,293 nucleotides, starting at the adenine (A) base located at position 109 within the 2nd exon and terminating at the adenine (A) base at position 1233 of the 26th exon. The DAPK1 protein, which consists of 1,430 amino acids, bears the following structure, from the N-terminus to the C-terminus (**Figure 1**): a kinase domain, a calmodulin (CaM) regulatory domain, a region of ankyrin repeats comprising eight repeats, a Ras of complex (ROC-COR) domain, a death domain (DD), as well as a serine-rich tail (18).

**Figure 2:**
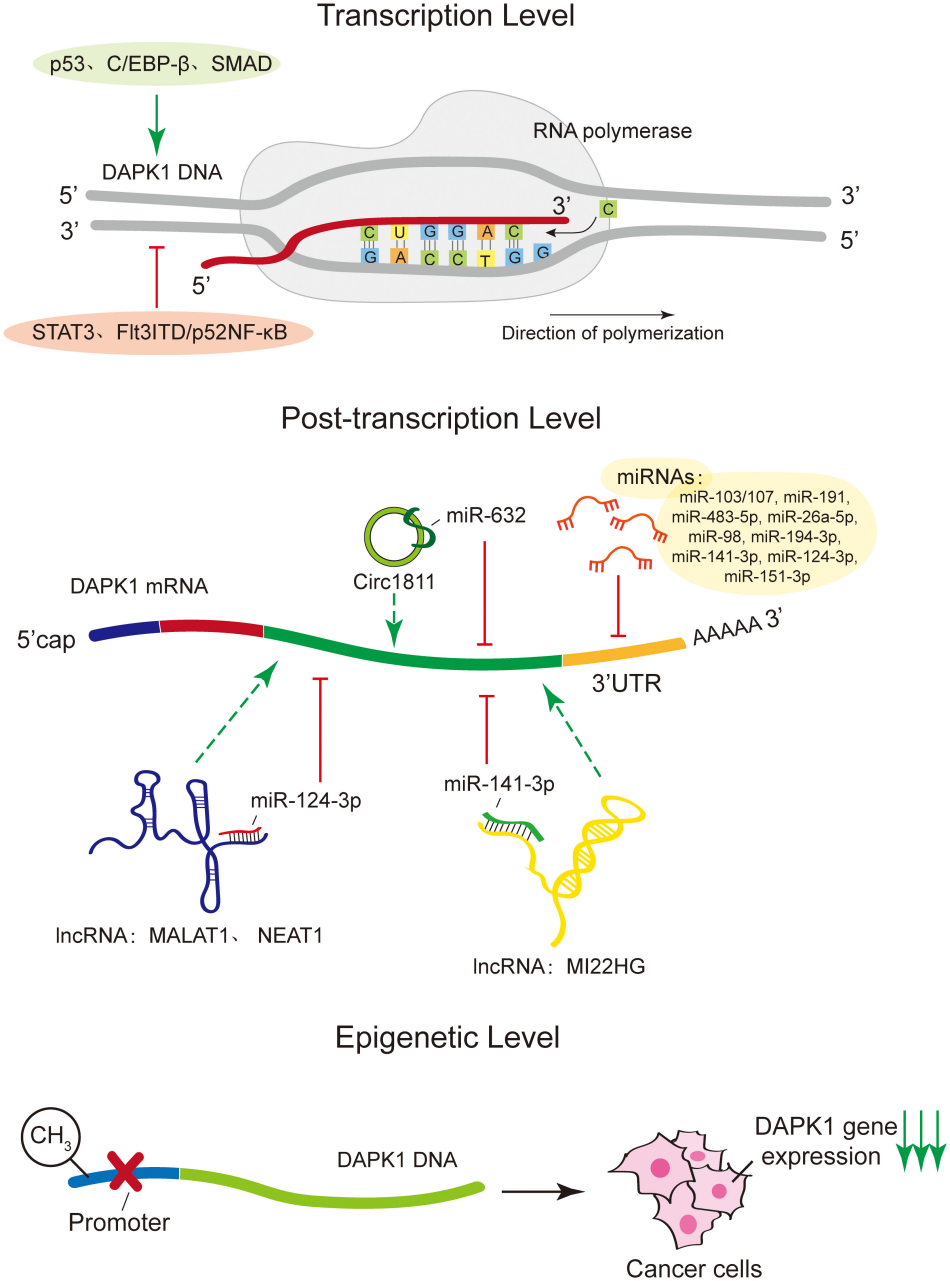
Regulatory mechanisms of DAPK1 expression. This figure illustrates the intricate regulation of DAPK1 expression at multiple levels. At the transcriptional level, DAPK1 is positively regulated by transcription factors such as p53, C/EBP-β, and SMAD, while STAT3 and Flt3ITD/p52NF-κB act as negative regulators. Post-transcriptional regulation involves various microRNAs (miRNAs) that downregulate DAPK1 mRNA expression by binding to its 3’ UTR, inhibiting translation or promoting mRNA degradation. These miRNAs include miR-103/107, miR-191, miR-483-5p, miR-26a-5p, miR-98, miR-194-3p, miR-141-3p, miR-124-3p, and miR-151-3p. Additionally, circRNA Circ1811 can sponge miR-632, thereby upregulating DAPK1 expression in gastric cancer cells. Long non-coding RNAs (lncRNAs) such as MALAT1, NEAT1, and MIR22HG also regulate DAPK1 expression by targeting specific miRNAs. Finally, DNA methylation was shown to suppress DAPK1 gene expression, particularly in cancer cells.

DAPK1 expression is intricately regulated at multiple levels (**Figure 2**). At the transcription level, a variety of transcription factors have been identified to modulate DAPK1 gene expression. Notably, tumor suppressor protein p53 (19), CCAAT/enhancer binding protein beta (C/EBP-β) (20) and small mother against decapentaplegic (SMAD) (21) act as positive transcriptional regulators, whereas signal transducer and activator of transcription 3 (STAT3) (22) and Fms-like tyrosine kinase 3 internal tandem duplication/p52 nuclear factor-kappa B (Flt3ITD/p52NF-κB) (23) function as negative regulators. Moreover, various microRNAs (miRNAs) have been reported to post-transcriptionally downregulate DAPK1 mRNA expression, including miR-103/107 (24), miR-191 (25), miR-483-5p (26), miR-26a-5p (27), miR-98 (28), miR-194-3p (29), miR-141-3p (30), miR-124-3p (31), and miRNA-151-3p (32) (**Table 1**). All of these miRNAs regulate DAPK1 mRNA levels through binding to its 3’ UTR, thus inhibiting the translation process or promoting mRNA degradation. In addition, in gastric cancer cells, Circ1811 can directly sponge miR-632, thereby preventing the inhibitory effect of miR-632 on DAPK1, and consequently upregulating the expression of DAPK1 (33). Furthermore, previous studies have indicated that long non-coding RNAs (lncRNAs) such as MALAT1 (31) and NEAT1 (34) upregulate DAPK1 expression by targeting miR-124-3p, and lncRNA MIR22HG also enhances DAPK1 levels by targeting miR-141-3p (35). Moreover, numerous studies have documented that DNA methylation suppresses DAPK1 gene expression, particularly in cancer cells (36, 37).

**Table 1:** Non-coding RNAs in the regulation of DAPK1.

Non-coding RNAs	Disease	Regulation	Ref
miR-103/107	Colorectal cancer	Down	(24)
miR-191	Sepsis-associated acute lung injury	Down	(25)
miR-483-5p	Nasopharyngeal carcinoma	Down	(26)
miR-26a-5p	Glioma	Down	(27)
miR-98	Cardiac ischemia	Down	(28)
miR-194-3p	Chronic obstructive pulmonary disease	Down	(29)
miR-141-3p	Polycystic ovary syndrome	Down	(30)
miR-124-3p miRNA-151-3p	Parkinson’s Disease AD	Down Down	(31, 32)
Circ1811/miR-632	Gastric cancer	Up	(33)
LncRNA MALAT1	Parkinson’s Disease	Up	(31)
LncRNA NEAT1	Cataract	Up	(34)
LncRNA MIR22HG	Endometrial carcinoma	Up	(35)

**Figure 3:**
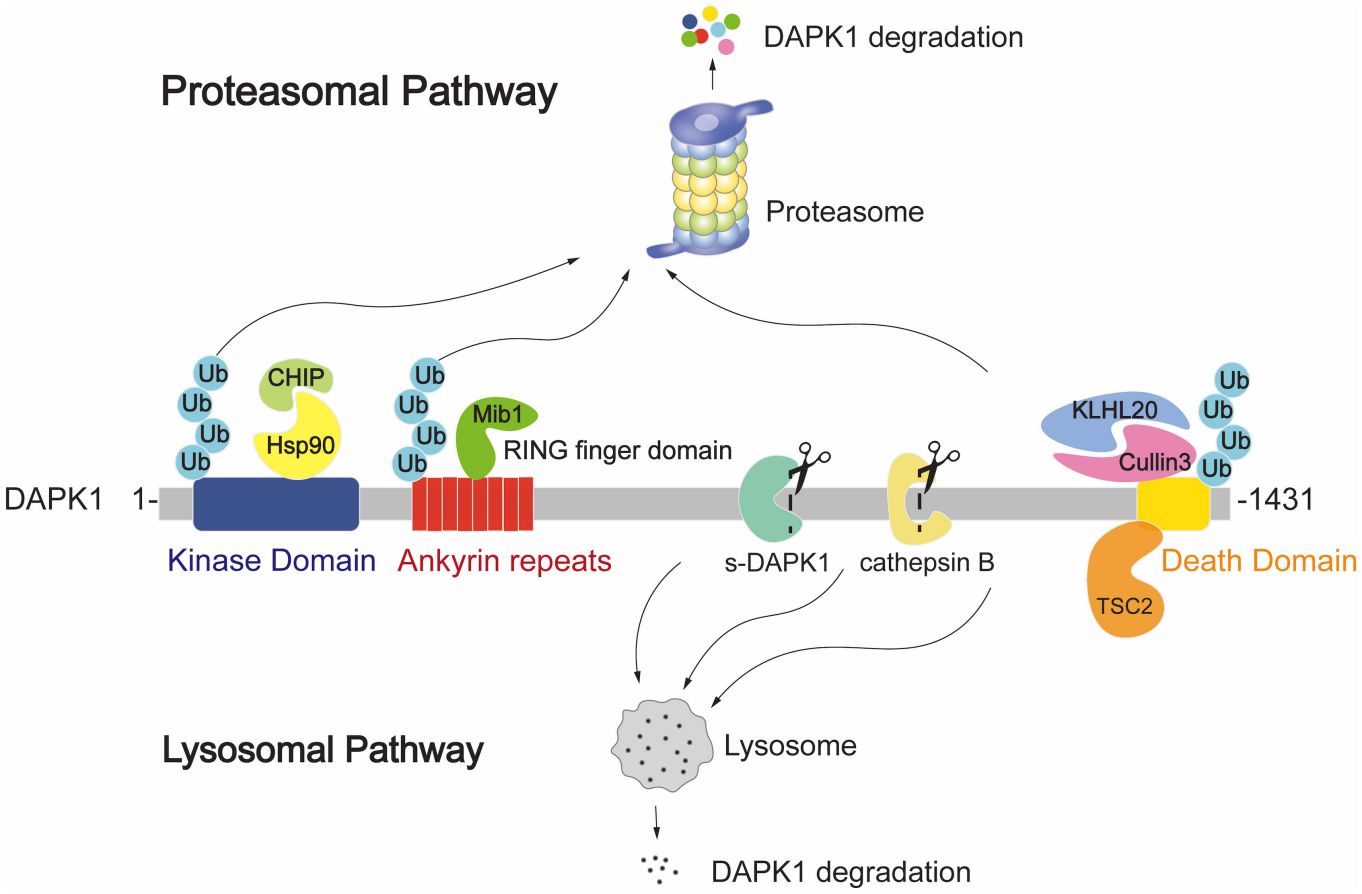
Illustration of the proteasomal and lysosomal DAPK1 degradation pathways. The ubiquitin E3 ligases Mib1, CHIP, and Cullin3 are known to regulate DAPK1 protein levels. Mib1 interacts with the ankyrin repeat region of DAPK1 via its RING finger domain. CHIP engages with the kinase domain of DAPK1 indirectly through its U-box domain’s interaction with Hsp90. Cullin3 forms a complex with KLHL20, which binds to the DD of DAPK1. TSC2 is depicted as facilitating lysosomal degradation of DAPK1 by binding to its DD through the C-terminus of TSC2. Additionally, the splice variant s-DAPK1 is shown to mediate lysosomal degradation of DAPK1, and the lysosomal protease cathepsin B is known to cleave DAPK1.

At the protein level, DAPK1 is subject to degradation through both the proteasomal and lysosomal pathways. The known ubiquitin E3 ligases that regulate DAPK1 protein levels include Mind bomb E3 ubiquitin protein ligase 1 (Mib1) (38), carboxyl terminus of HSC70-interacting protein (CHIP) (39) and Cullin3 (40). These E3 ligases are involved in the proteasomal degradation pathway of DAPK1, as illustrated in **Figure 3**, where the ubiquitination and subsequent degradation of DAPK1 are depicted. Mib1 interacts with the ankyrin repeat region of DAPK1 via its RING finger domain, while CHIP engages with the kinase domain of DAPK1 indirectly via its U-box domain’s interaction with heat shock protein 90 (Hsp90) (41) (**Figure 3**, left panel). On the other hand, Cullin3 forms a complex with KLHL20, which binds to the DD of DAPK1 (42) (**Figure 3**, right panel). However, despite these reports of the ubiquitin E3 ligases of DAPK1, the specific ubiquitination sites of DAPK1 remain to be identified. It would be intriguing to explore whether these E3 ligases target the same lysine residues for ubiquitination and to compare the interactions and crosstalk among them in the context of DAPK1 ubiquitination. Moreover, as shown in **Figure 3**, TSC complex subunit 2 (TSC2) has been reported to facilitate the lysosomal degradation of DAPK1 by binding to the DD of DAPK1 through its C-terminus (43). However, the precise molecular mechanism underlying TSC2-mediated DAPK1 degradation remains to be elucidated. Additionally, a splice variant of DAPK1 (s-DAPK1) has been shown to mediate lysosomal degradation of DAPK1, and the lysosomal protease cathepsin B was found capable of cleaving DAPK1 (44) (**Figure 3**).

Post-translational modifications of DAPK1, particularly phosphorylation, play a crucial role in regulating its activity, stability, and interactions with other proteins. Phosphorylation at Ser308, located within the Ca^2+^-calmodulin-binding autoinhibitory domain, is one of the most studied modifications of DAPK1. This phosphorylation inhibits DAPK1’s catalytic activity by stabilizing its autoinhibitory conformation, preventing calmodulin from fully activating the kinase. For instance, GTP binding to the ROC domain of DAPK1 has been shown to enhance autophosphorylation at Ser308, which contributes to turning the kinase to the ‘off’ state (45). In cervical cancer cells, DAPK1 is autophosphorylated at Ser308 during the G2 phase and mitosis, and this phosphorylation is further regulated by Polo-like Kinase 1 (PLK1), a key mitotic regulator, particularly during the G2/M phase (46). A deletion mutation of the Ca^2+^-CaM binding domain has a constitutive activation effect on its kinase activity (18, 47). Dephosphorylation of Ser308, often mediated by phosphatases such as Protein Phosphatase 2A (PP2A), results in the activation of DAPK1, triggering downstream signaling pathways involved in apoptosis and autophagy (48–50). Furthermore, phosphorylation of Tyr491 and Tyr492 is critical for DAPK1 activation (51, 52). These residues are located within the catalytic domain and help stabilize the kinase in an active conformation (52). Src family kinases, such as Src and Fyn, are known to phosphorylate DAPK1 at these sites, enhancing its pro-apoptotic activity (52). Phosphorylation at Ser734 has been shown to modulate the interaction of DAPK1 with other signaling proteins and regulate its subcellular localization (53). Extracellular signal-regulated kinase (ERK) interacts with DAPK1 via the DD of DAPK1 and directly phosphorylates it at Ser735 (54). This phosphorylation enhances the catalytic activity of DAPK1, thereby promoting its pro-apoptotic function (54). In contrast, Ras-ERK activation, mediated through p90 ribosomal S6 kinase (RSK), phosphorylates DAPK1 at Ser289, thereby suppressing its apoptotic activity (55).

## 2 Cellular functions of DAPK1

**Figure 4:**
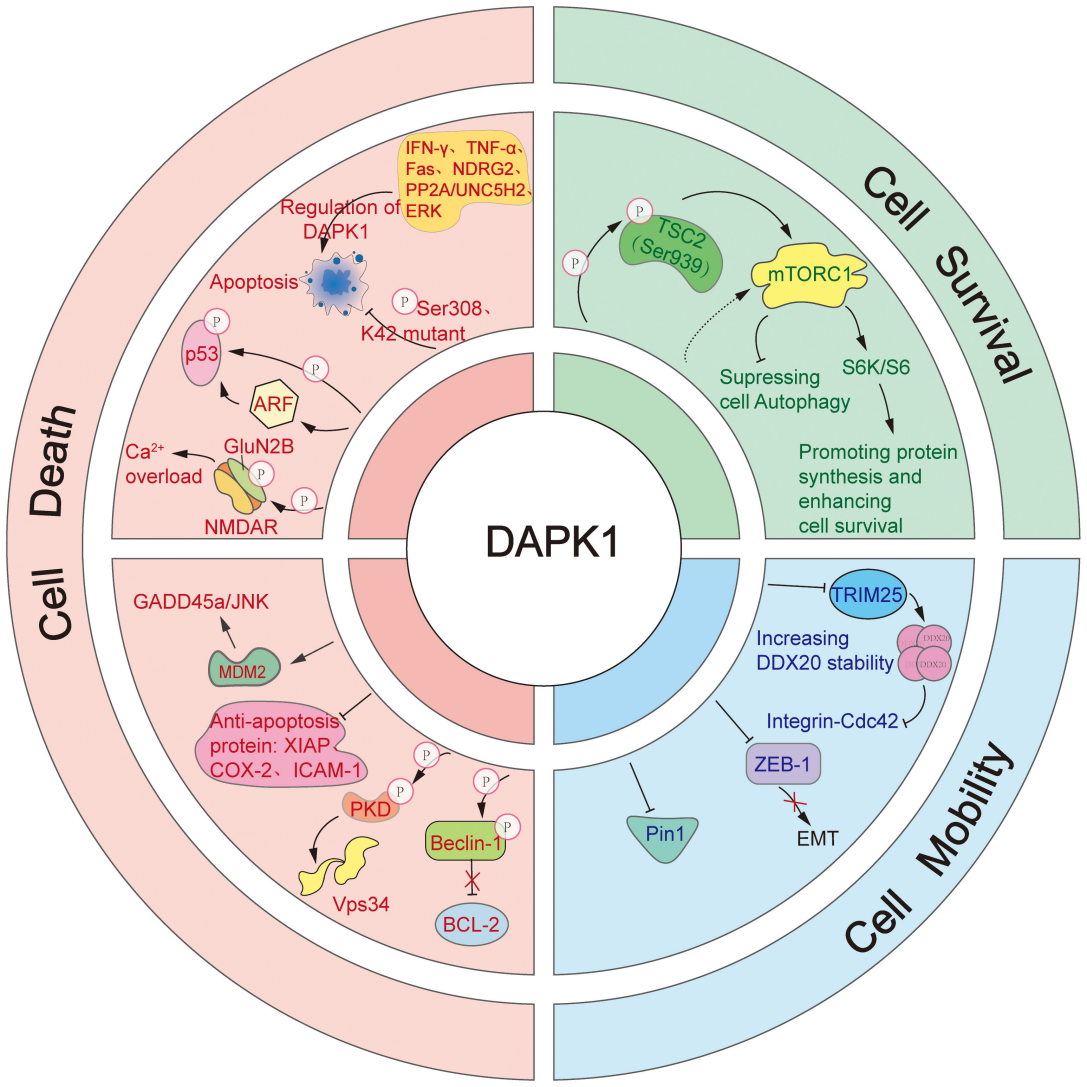
The diverse cellular functions of DAPK1.This figure illustrates the complex and multifaceted roles of DAPK1 in regulating cell death, cell survival, and cell mobility. DAPK1 is a versatile inducer of apoptosis, activated by various upstream signals such as IFN-γ, TNF-α, Fas, PP2A/UNC5H2, and NDRG2. It promotes apoptosis through multiple downstream pathways, including direct phosphorylation of p53, activation of ARF, suppression of anti-apoptotic proteins (e.g., XIAP, COX-2, ICAM-1), activation of the GADD45a/JNK pathway via MDM2 regulation, and enhancement of Ca^2+^ influx by phosphorylating GluN2B. Additionally, DAPK1 induces autophagic cell death by phosphorylating PKD to activate Vps34 or by directly phosphorylating Beclin 1 to release Bcl-2. In contrast, DAPK1 also supports cell survival by activating mTORC1 through TSC2 phosphorylation, which stimulates p70 S6K and S6, enhancing protein synthesis. Furthermore, DAPK1 regulates cell mobility by inhibiting the integrin-mediated polarity pathway via TRIM25-mediated degradation of DDX20, suppressing EMT induced by ZEB1, and inhibiting the activity of Pin1.

DAPK1 is a multifunctional protein kinase that has garnered significant attention due to its diverse and often opposing roles in cellular processes. This section will provide a comprehensive overview of the cellular functions of DAPK1, focusing on its roles in cell death, cell survival, and cell mobility (**Figure 4**). These functions are intricately regulated by various signaling pathways and molecular interactions, highlighting the complexity and versatility of DAPK1 in cellular physiology and pathology.

### 2.1 Cell death

DAPK1 is a versatile inducer of cell death, with a primary focus on apoptosis. It acts as a positive regulator of apoptosis in response to a variety of upstream signals, including IFN-γ (1), tumor necrosis factor-alpha (TNF-α) (54), fas cell surface death receptor (Fas) (56), PP2A/Netrin-1 receptor uncoordinated protein 5 homolog 2 (PP2A/UNC5H2) (57), and n-myc downstream regulated gene2 (NDRG2) (58). The kinase activity of DAPK1 is pivotal for its pro-apoptotic role. Autophosphorylation at Ser308 or mutation at the K42 site reduces its kinase activity, thereby inhibiting its pro-apoptotic function (18, 59). In contrast, activation of its catalytic activity by ERK enhances its pro-apoptotic capabilities (60). The DD of DAPK1 is also crucial for its pro-apoptotic function, as co-transfection with the DD can mitigate apoptosis induced by DAPK1 overexpression (56).

DAPK1 can stimulate apoptosis via multiple downstream pathways. DAPK1 has been reported to trigger p53-mediated apoptosis either by directly phosphorylating p53 at the Ser23 residue or by activating ADP Ribosylation Factor (ARF) (61, 62). Moreover, DAPK1 is known to suppress the expression of anti-apoptotic proteins such as X-linked inhibitor of apoptosis protein (XIAP), cyclooxygenase-2 (COX-2), and intercellular adhesion molecule-1 (ICAM-1) (63). Additionally, DAPK1 can activate the growth arrest and DNA damage inducible alpha/c-Jun N-terminal kinase (GADD45a/JNK) pathway by regulating murine double minute 2 (MDM2), thereby mediating apoptosis (64). DAPK1 also enhanced Ca^2+^ influx by phosphorylating N-methyl-D-aspartate (NMDA) receptor subunit GluN2B (GluN2B), leading to neuronal cell apoptosis (65). Consistently, it was further found that inhibition of DAPK1 activity, knockdown of DAPK1 expression, and antagonism of GluN2B subunits, effectively prevented activation of GluN2B subunit, Ca^2+^ overload, and neuronal apoptosis.

Beyond apoptosis, enforced expression of DAPK1 was shown to induce autophagic cell death as well (66–68). Mechanistically, DAPK1 can induce autophagic cell death in two distinct ways. Firstly, it phosphorylates protein kinase D (PKD) to activate phosphatidylinositol 3-kinase catalytic subunit type 3 (Vps34) (66). Secondly, it directly phosphorylates Beclin 1, a component of the Vps34 complex, leading to the release of Bcl-2 (67, 69). These mechanisms highlight the central role of DAPK1 in regulating cell death pathways, showcasing its adaptability in modulating cell death in response to various stimuli.

### 2.2 Cell survival

Our previous research has uncovered DAPK’s ability to activate the mechanistic target of rapamycin complex 1 (mTORC1) by suppressing TSC2 (70). Since the activation of mTORC1 is known to inhibit autophagy, our findings suggest that DAPK may also play a role in suppressing this process. The effect of DAPK1 on autophagy could be dependent on the specific stimuli it encounters. Furthermore, DAPK1 has been shown to phosphorylate TSC2 at Ser939, which in turn activates mTORC1 (70, 71). This activation by DAPK1 subsequently leads to stimulation of p70 ribosomal protein S6 kinase (S6K) and ribosomal protein S6 (S6), promoting protein synthesis and enhancing cell survival (43).

### 2.3 Cell mobility

DAPK1 has been identified as a multifaceted regulator of cell mobility, employing several strategies to suppress it. Firstly, DAPK is known to inhibit cell mobility by obstructing the integrin-mediated polarity pathway, which is crucial for cell movement and orientation (72). Our recent publications have elucidated that DAPK1 curbs the integrin- cell division cycle 42 (Cdc42) polarity pathway through the inhibition of tripartite motif containing 25 (TRIM25)-mediated proteasomal degradation of DEAD-box decapping enzyme 20 (DDX20), a key player in cellular polarity and migration (73, 74). Secondly, DAPK1 has been reported to suppress epithelial-mesenchymal transition (EMT) induced by zinc finger E-box binding homeobox protein 1(ZEB1), a transcriptional repressor that promotes cell mobility, thereby inhibiting cell movement (75). Thirdly, DAPK1 is known to inhibit peptidylprolyl isomerase NIMA-interacting 1 (Pin1), a peptidyl-prolyl isomerase that is pivotal for numerous cellular functions, including cell mobility (76, 77).

## 3 The role of DAPK1 in malignant and non-malignant diseases

DAPK1 has been implicated in a wide range of diseases, both malignant and non-malignant. Its diverse roles in cellular processes such as apoptosis, autophagy, and cell mobility make it a key player in the pathogenesis of various disorders. This section will provide an in-depth exploration of DAPK1’s involvement in different diseases, including cancer, stroke, neurodegenerative diseases, cardiovascular diseases, wound healing, kidney injury, and tuberous sclerosis complex (**Table 2**). The discussion will highlight the complex and often contradictory roles of DAPK1 in these conditions, emphasizing the need for further research to elucidate its mechanisms and potential as a therapeutic target.

**Table 2:** The role of DAPK1 in malignant and non-malignant diseases.

Type	Disease Type	Mechanisms	Clinical Role	Ref
Cancer	B-cell malignancies	Hypermethylation of the DAPK1 promoter leads to downregulation of DAPK1 mRNA expression	Tumor suppressor gene	(78)
	Breast cancer	Promoter methylation of the DAPK1 gene was not significantly correlated with DAPK1 protein	Tumor suppressor gene	(79)
	p53-mutant breast cancer	Promotes cell survival via phosphorylation of TSC2 at Ser939 and activation of the mTORC1 pathway	Oncogene	(80)
	Gastric cancer	High DAPK1 expression promotes metastasis;	Oncogene	(81)
		As a downstream target of tumor suppressor Circ1811, inducing IFN-γ induced cell death, or mediating IKKβ/CSN5/PD-L1 axis to enhance natural killer cell killing and inhibit tumor immune evasion	Tumor suppressor gene	(33, 82)
Stroke/Brain damage	Ischemic stroke	Mediates apoptosis and autophagy, recruited to NMDA receptor NR2B subunit, enhancing receptor activity and leading to excitotoxicity, facilitates injurious calcium influx	Exacerbates neuronal damage	(83–85)
	Ischemic stroke	Modulates the ERK/CREB/BDNF signaling pathway, participates in autophagy, activity modulated by interaction with LRRFIP1	Increases neuronal apoptosis, cognitive dysfunction, contributes to cell death	(86–88)
Neurodegenerative diseases	AD	Contributes to excitotoxicity by phosphorylating NMDA receptor subunit GluN2B	Leads to calcium overload and neuronal cell apoptosis	(65, 89)
	Neurological disorders	Activates death signaling pathways	Regulates apoptotic neuronal cell death	(90)
Cardiovascular diseases	Acute myocardial infarction	Promotes inflammation and oxidative damage	DAPK1 inhibitor plays a protective role	(91, 92)
	Hypertension	Enhances vasoconstriction through myosin light chain phosphorylation	Regulates blood pressure	(93)
Wound healing	Skin wounding	Negatively regulates calcium-dependent signaling cascade promoting wound closure, modulates actin cytoskeleton and cellular motility	Negative regulator	(7, 94)
	Wound healing	Influences outcomes through apoptosis and cell turnover regulation during tissue remodeling	Pro-apoptotic functions	(95–97)
Kidney injury	Acute kidney injury	Destabilizes Pellino1 via recruiting caspase-8 with TRIF-RIP1 signalosome, leading to Pellino1 poly-ubiquitination and turnover	Mediate tubular damage	(98)
	Ischemic kidney injury	Promotes cell death signaling and inhibits neural remodeling	Negative regulator	(99)
tuberous sclerosis complex	Tuberous sclerosis complex	May interact with the mTOR pathway and influence the development of TSC-related lesions	NA	(43, 71, 99)

NA, Not available.

### 3.1 Cancer

DAPK1 is well-known as a tumor suppressor gene due to its cell death inducer function (100). In 1997, promoter hypermethylation of DAPK1 was first reported in B-cell malignancies, which lead to loss of DAPK1 mRNA expression (78). Since then, over 200 papers studied the hypermethylation of the DAPK1 promoter in various tumors. It is natural to infer that hypermethylation of DAPK1 not only silences its gene expression but also contributes to the malignancy state of tumors, making it a potential biomarker for cancer diagnosis and prognosis (101, 102). However, most studies only examined the promoter methylation status of the DAPK1 gene, and not many studies thoroughly investigated the mRNA and protein levels of DAPK1. Our previous studies with breast cancer specimens demonstrated that the methylation of DAPK1 gene did not correlate well with DAPK1 protein level (79). Therefore, it is worthwhile investigating whether DAPK1 promoter methylation may reflect other biological readouts apart from DAPK1 level, such as the activity of relevant methyltransferases.

**Figure 5:**
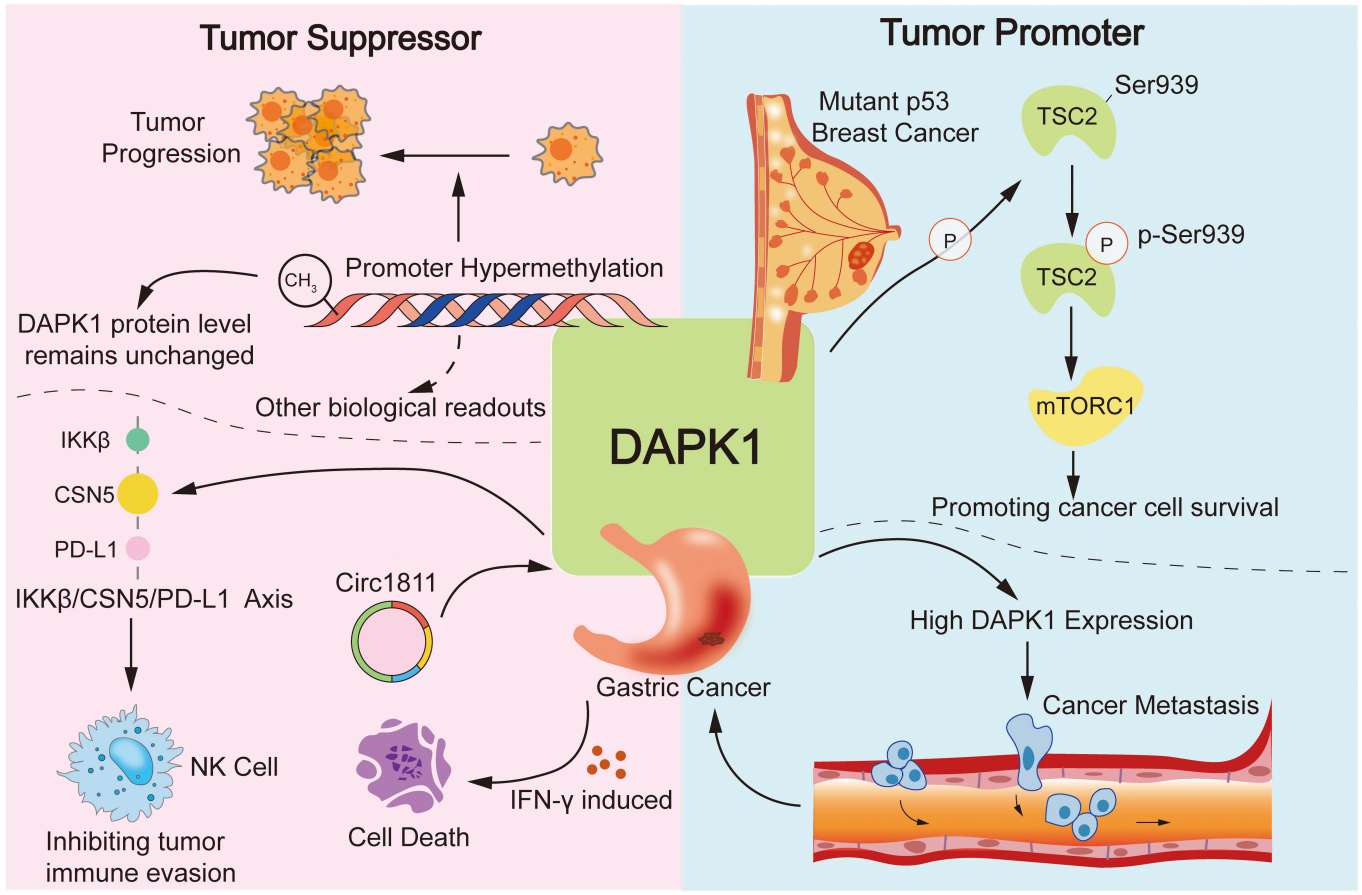
Dual roles of DAPK1 in cancer. This figure illustrates the complex and dual roles of DAPK1 in cancer, highlighting its functions as both a tumor suppressor and an oncogene. DAPK1 is a well-known tumor suppressor due to its ability to induce cell death, with promoter hypermethylation leading to loss of its expression in various cancers. However, DAPK1 also acts as an oncogene in certain contexts, such as promoting p53-mutant breast cancer cell survival via mTORC1 activation and enhancing gastric cancer metastasis. Conversely, DAPK1 can act as a tumor suppressor in gastric cancer by mediating the IKKβ/CSN5/PD-L1 axis to inhibit immune evasion.

Moreover, DAPK1 was also reported as an oncogene in some other studies. For instance, DAPK1 was found to promote p53-mutant breast cancer cell survival via phosphorylation of TSC2 at Ser939 and subsequent activation of the mTORC1 pathway (80). Our previous study also discovered that high DAPK1 expression promotes gastric cancer metastasis (81). However, some recent studies suggested the opposite and demonstrated that DAPK1 can act as a tumor suppressor in gastric cancer either via as a downstream target of tumor suppressor Circ1811, instigating IFN-γ induced cell death, or mediating IKKβ/CSN5/PD-L1 axis to enhance natural killer cell killing and inhibiting tumor immune evasion (**Figure 5**) (33, 82). The contradictory reports were not only evident in gastric cancer, but also in many other cancers such as hepatocellular carcinoma (HCC), breast cancer, etc. (73, 103–107). Hence, more in-depth research systems are needed to clarify these confusing studies in various cancers.

### 3.2 Stroke/brain damage

DAPK1 plays a significant role in the pathophysiology of stroke, primarily through its involvement in neuronal cell death pathways (83). DAPK1 is known to mediate apoptosis and autophagy, processes that are critical in the context of ischemic stroke (84). During a stroke, DAPK1 is recruited to the NMDA receptor NR2B subunit, enhancing receptor activity and leading to excitotoxicity, a major cause of neuronal cell death (84, 85). This key interaction facilitates injurious calcium influx, exacerbating neuronal damage (84).

Moreover, the role of DAPK1 extends beyond direct interaction with NMDA receptors. It is involved in the regulation of various intracellular signaling pathways that contribute to cell death and survival. For instance, DAPK1 has been implicated in the modulation of the ERK/CREB/BDNF signaling pathway, which is crucial for neuronal survival and cognitive functions (86). Dysregulation of this pathway by DAPK1 can lead to increased neuronal apoptosis and cognitive dysfunction, particularly in post-stroke conditions (86).

In addition to its role in apoptosis, DAPK1 is also involved in autophagy, a process that can play both protective and detrimental functions depending on the context. In the setting of ischemic stroke, DAPK1-mediated autophagy may contribute to cell death, further highlighting its complex role in stroke pathology (87). The kinase’s activity is modulated by various factors, including its interaction with other proteins such as Leucine-Rich Repeat Flightless-1 Interacting Protein 1(LRRFIP1), which has been identified as a novel interactor in stroke-like conditions (88).

Therapeutically, targeting DAPK1 presents a promising strategy for neuroprotection in stroke. Inhibition of DAPK1 activity has been shown to reduce neuronal cell death and improve outcomes in experimental models of stroke (108). This is supported by studies demonstrating that pharmacological or genetic blockade of DAPK1 can attenuate excitotoxicity and protect against ischemic brain injury (109). Furthermore, the development of specific DAPK1 inhibitors that disrupt its interaction with the NMDA receptor NR2B subunit is an area of active research, with potential implications for reducing stroke-induced neuronal cell damage (110).

Collectively, DAPK1 is a critical mediator of neuronal cell death in stroke, acting through multiple pathways to influence cell fate. Its modulation offers a potential therapeutic avenue for mitigating the detrimental effects in stroke and improving patient outcomes.

### 3.3 Neurodegenerative diseases

Neuronal cell death that DAPK1 is implicated in is a critical process in the pathogenesis of various neurodegenerative diseases (83). In the context of neurodegenerative disorders, DAPK1 has been shown to play a significant role in Alzheimer’s disease (AD) by contributing to excitotoxicity, a process where excessive stimulation by neurotransmitters such as glutamate leads to neuronal cell injury and death (89). The phosphorylation of the NMDA receptor subunit GluN2B by DAPK1 is one mechanism through which excitotoxicity is mediated in AD, leading to calcium overload and neuronal cell apoptosis (65).

Furthermore, DAPK1’s involvement in neurodegenerative diseases extends beyond AD (90). Its deregulation has been associated with the progression of other neurological disorders, where it activates death signaling pathways and regulates apoptotic neuronal cell death (90). The phosphorylation-dependent and independent pathways of DAPK1 have been studied to understand its role in neuronal apoptosis under various stress conditions (83). This understanding is crucial for developing therapeutic strategies targeting DAPK1 to mitigate its detrimental effects in neurodegenerative diseases (83).

Research has also highlighted the potential of targeting DAPK1 for therapeutic intervention. By inhibiting DAPK1 activity or expression, it is possible to prevent the downstream effects that lead to neuronal damage. This approach could be beneficial in treating diseases where DAPK1 plays a pivotal role in disease progression. The development of drugs that specifically target DAPK1 signaling pathways could offer new avenues for the treatment of neurodegenerative diseases, providing hope for conditions that currently have limited therapeutic options (65, 83).

### 3.4 Cardiovascular diseases

DAPK1 has emerged as a significant player in the pathogenesis of heart disease, particularly in the context of myocardial infarction and heart failure (111). In the former context, DAPK1 inhibitor has been shown to protect against myocardial injury by inhibiting inflammation and oxidative stress (91, 92). In a study using a rat model of acute myocardial infarction (AMI), the DAPK1 expression was significantly higher in AMI rats compared to controls (91). The use of a DAPK1 inhibitor reduced the expression of inflammatory factors and oxidative stress markers, suggesting that DAPK1 inhibitor plays a protective role in cardiomyocytes by mitigating inflammation and oxidative damage (91). Moreover, our recently published study demonstrates that DAPK1 promotes hypertension by enhancing vasoconstriction through myosin light chain phosphorylation, and its inhibition effectively attenuates hypertension and associated vascular damage (93).

### 3.5 Wound healing

The functional role of DAPK1 in wound healing is an intriguing research area, particularly due to its involvement in regulating cellular processes such as apoptosis, autophagy, and cytoskeletal dynamics (94–96, 112). In the context of wound healing, DAPK1 has been shown to act as a negative regulator of wound closure (7, 94). Research using the model organism Caenorhabditis elegans has demonstrated that skin wounding triggers a calcium-dependent signaling cascade that promotes wound closure, which is negatively regulated by DAPK1 (94). This suggests that DAPK1 may play a role in modulating the actin cytoskeleton and cellular motility, which are crucial for effective wound repair. Furthermore, the involvement of DAPK1 in apoptosis could also influence wound healing outcomes (96). Apoptosis is a critical process during the remodeling phase of wound healing, where the removal of excess cells is necessary for proper tissue regeneration (95). DAPK1 pro-apoptotic functions may therefore contribute to the regulation of cell turnover and tissue remodeling during the healing process (95, 97). Additionally, the interaction of DAPK1 with other signaling pathways, such as the MAPK pathway, could further elucidate its role in wound healing (94). The MAPK pathway is known to be involved in various stages of wound repair, including inflammation, proliferation, and remodeling (113–115). Understanding how DAPK1 interacts with these pathways could provide insights into its comprehensive role in wound healing dynamics.

Overall, while the precise mechanisms by which DAPK1 influences wound healing are still being elucidated, its regulatory functions in apoptosis, cytoskeletal dynamics, and interaction with signaling pathways suggest that it plays a significant role in the wound healing process. Further research into DAPK1 could potentially lead to novel therapeutic strategies for enhancing wound repair and managing chronic wounds.

### 3.6 Kidney injury

The role of DAPK1 in kidney injury, particularly in acute kidney injury (AKI) and chronic kidney disease (CKD), has been a subject of extensive research (98, 116). DAPK1 is known to interact with several signaling pathways that contribute to kidney damage, making it a potential therapeutic target (98, 116, 117). In the context of AKI, DAPK1 has been shown to mediate tubular damage through its involvement in apoptotic and inflammatory pathways (98). DAPK1 destabilizes Pellino1, which governs inflammation-coupling tubular damage during septic AKI, via recruiting caspase-8 with TRIF-RIP1 signalosome, leading to Pellino1 poly-ubiquitination and turnover (98). Inhibition or genetic ablation of DAPK1 has been found to protect tubular cells from LPS-induced damage under hypoxic conditions, suggesting that targeting DAPK1 could be a viable strategy for managing septic AKI (98). Moreover, the suppression of DAPK1 has been shown to reduce ischemic brain injury by inhibiting cell death signaling and promoting neural remodeling, which could have parallels in renal tissue, suggesting a potential for therapeutic interventions targeting DAPK1 in ischemic kidney injury (99). These findings suggest that DAPK1 may similarly influence inflammatory and oxidative pathways in kidney injury, providing further rationale for its targeting in renal pathologies (91).

Overall, DAPK1 plays a multifaceted role in kidney injury, influencing apoptotic, inflammatory, and oxidative stress pathways. Its modulation presents a promising avenue for therapeutic intervention in both acute and chronic kidney diseases.

### 3.7 Tuberous sclerosis complex

DAPK1 has been implicated in various cellular processes, and its role in TSC is an area of active research (43, 118). TSC is a rare multisystem autosomal dominant genetic disease characterized by benign tumors growing in the brain, spinal cord, nerves, but also in kidneys, heart, liver, lungs, eyes, and skin. TSC is caused by loss of function mutations in TSC1 or TSC2 genes (119), leading to dysregulation of the mTOR signaling pathway (120). While the primary focus has been on mTOR inhibitors as a therapeutic strategy, recent studies suggest that DAPK1 may also play a significant role in the pathogenesis of TSC. In the context of TSC, DAPK1 has been shown to interact with the mTOR pathway, potentially influencing the development of TSC-related lesions (43). Research indicates that DAPK1 may contribute to the regulation of cell death and survival pathways, which are critical for the formation of hamartomas, a hallmark of TSC (71, 99). Inhibition of DAPK1 has been associated with reduced cell death signaling and enhanced neural remodeling, suggesting a potential therapeutic target for TSC-related neurological manifestations (99).

Moreover, studies have demonstrated that DAPK1 can modulate the apoptotic pathways in various cellular contexts (19, 61). For instance, in breast cancer, DAPK1 expression is often downregulated, and its methylation status is altered, indicating its role in tumorigenesis (121). This highlights the broader implications of DAPK1 in disease mechanisms beyond TSC, suggesting that its regulation could be pivotal in managing TSC-related symptoms. Additionally, the interplay between DAPK1 and other signaling molecules, such as BAX/BCL2 and LC3/Beclin1, underscores its involvement in apoptosis and autophagy, processes that are crucial in the cellular environment of TSC lesions (99). The potential of DAPK1 as a therapeutic target is further supported by findings that its suppression can lead to improved outcomes in ischemic brain injury models, which may parallel the neural damage observed in TSC (99).

In conclusion, while the mTOR pathway remains a central focus in TSC research, the role of DAPK1 offers a promising avenue for therapeutic intervention. By targeting DAPK1, it may be possible to modulate cell death and survival pathways, thereby alleviating some of the neurological and systemic manifestations of TSC. Further research is needed to fully elucidate the mechanisms by which DAPK1 influences TSC pathology and to develop targeted therapies that can effectively mitigate its impact.

## 4 Targeting DAPK1 by small molecule inhibitors for the treatment of human diseases

Recent studies have explored the potential of small molecules to modulate DAPK1, offering new avenues for treating malignant as well as non-malignant disorder like cancer, neurodegenerative disorders, and cardiovascular diseases. In this section, we summarize the published small molecules that exert therapeutic effects on human diseases via multiple mechanisms regulating DAPK1 (**Table 3**). The inhibitors are categorized based on their specificity and potency, with clear distinctions between those with well-defined mechanisms and those with less clear mode of action.

**Table 3:** Small molecules in regulation of DAPK1.

Name/Function	Specificity; Effect on DAPK1	Dose	Cellular effect	Disease	Ref
Panobinostat/LBH589	Non-specific; protein enhanced; Ser308 dephosphorylation	0.05 μM	Induce autophagy; colorectal cancer cells	Colorectal cancer cells	(122)
Trichostatin A	Non-specific; protein enhanced; Ser308 dephosphorylation	IC_50_ = 418.7 ± 25.12 nM; IC_50_ = 446.6 ± 27.32 nM	Induces apoptosis; lung cancer cells	Lung cancer cells	(123)
Sodium butyrate	Non-specific; mRNA & protein enhanced	2 μM; 3 mM	Induces apoptosis; gastric cancer cells; Raji cells	Gastric cancer cells; Raji cells	(124, 125)
Vidaza (5-azacytidine)	Non-specific; mRNA enhanced	5 μM	Induces apoptosis; breast tumors; colorectal and gastric cancers; lung cancers.	Canine mammary gland tumor cells	(17)
Decitabine (5-aza-2’-deoxycytidine)	Non-specific; mRNA enhanced	IC_50_ = 5 μM	Induces apoptosis; breast tumors; colorectal and gastric cancers; lung cancers	Cholangiocarcinoma cells, chronic lymphocytic leukemia	(126, 127)
Sodium selenite	Non-specific; mRNA and protein enhanced; Ser308 dephosphorylation	20 μM	Induces autophagy; HL60 cells	HL60 cells	(128)
Curcumin	Non-specific; mRNA & protein enhanced	40 μM	G_2_/M arrest; apoptosis; (U251 cell)	Glioblastoma multiforme U251 cells	(129)
E-64d	Non-specific; protein enhanced	10 μg/mL	NA	NA	(43)
Chloroquine	Non-specific; protein enhanced	100 μM	NA	NA	(43)
Leupeptin	Non-specific; protein enhanced	200 μM	NA	NA	(43)
MG132	Non-specific; protein enhanced	10 μM	NA	NA	(43)
TC-DAPK6	Specific	IC_50_ = 69 nM	NA	Traumatic brain injury and related neurodegenerative disorders	(130)
HS38	Specific	IC_50_ = 200 nM	NA	AD and ischemic stroke	(131)
CPR005231	Specific	IC_50_ = 247 nM	NA	AD and ischemic stroke	(132)
Morin	Specific	IC_50_ = 11 μM	NA	Neuronal Cell Death and Neurodegenerative Disease, AD	(83, 133)
Isoliquiritigenin	Non-specific	30 μM; 90 μM	NA	AD and cerebral ischemia	(134)
Purpurin	Specific	IC_50_ = 0.89 mM	NA	NA	(135)
6-Shogaol	Non-specific	10 μM	NA	Ischemic stroke; cerebral ischemia-reperfusion injury	(136, 137)
Sanggenon C	Non-specific	10 μM; 20 μM	NA	Glioblastoma	(38)
SP600125	Non-specific	20 μM	NA	Neurodegenerative diseases and cancer	(138, 139)
SB203580	Non-specific; Ser308 dephosphorylation	20 μM; 5 mg/kg; 40 mg/kg	Induces autophagy	AD and ischemic stroke; mouse leukemia L1210/VCR cells	(140–142)
Grifolin	Non-specific; mRNA & protein enhanced; Ser308 dephosphorylation	30 μM; 40 μM	Induces apoptosis	Nasopharyngeal carcinoma cells	(143, 144)

NA, Not available; AD, Alzheimer’s disease.

### 4.1 Regulation of DAPK1 expression

**Figure 6:**
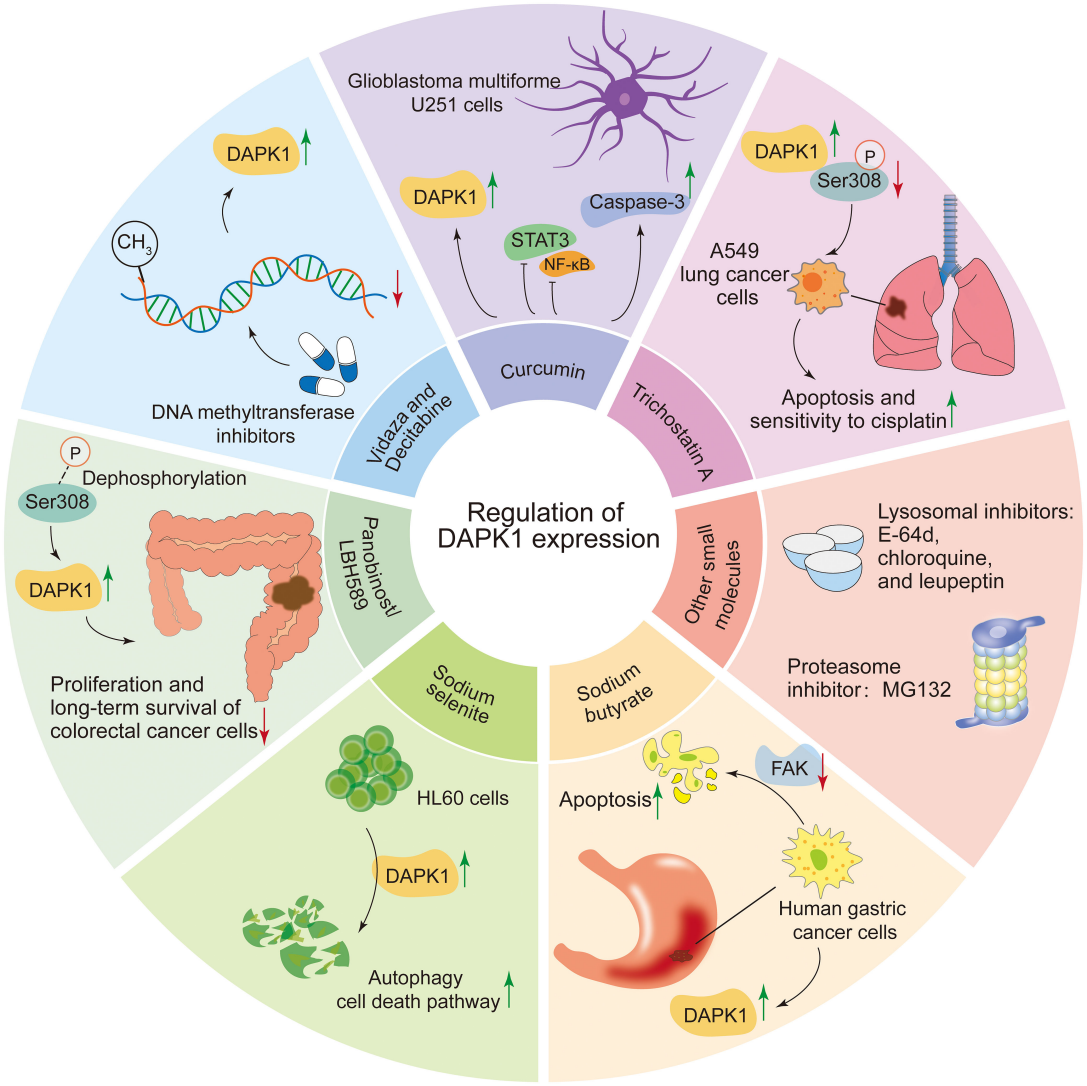
Compounds regulating DAPK1 expression. This figure summarizes 11 compounds reported to regulate DAPK1 expression, including Panobinostat/LBH589, Trichostatin A, sodium butyrate, 5-azacytidine (Vidaza), decitabine (5-aza-2’-deoxycytidine), sodium selenite, curcumin, E-64d, chloroquine, leupeptin, and MG132. LBH589 activates DAPK1 via Ser308 dephosphorylation, inducing autophagy in colorectal cancer cells. Trichostatin A upregulates DAPK1 and downregulates Ser308 phosphorylation, promoting apoptosis in A549 lung cancer cells. Sodium butyrate induces DAPK1 expression and reduces FAK levels, triggering apoptosis in gastric cancer and Raji cells. DNA methyltransferase inhibitors Vidaza and Decitabine restore DAPK1 expression by hypomethylating its promoter in various cancers. Sodium selenite activates DAPK1, triggering autophagy and cell death in HL60 cells. Curcumin upregulates DAPK1, inhibits STAT3 and NF-κB, and induces apoptosis in glioblastoma cells. Other regulators include lysosomal inhibitors (E-64d, chloroquine, leupeptin) and the proteasome inhibitor MG132.

A total of 11 compounds have been reported to regulate DAPK1 expression, including Panobinostat/LBH589, Trichostatin A, sodium butyrate, 5-azacytidine (Vidaza), decitabine (5-aza-2’-deoxycytidine), sodium selenite, curcumin, E-64d, chloroquine, leupeptin, and MG132 (17, 43, 122–129, 145–148) (**Figure 6**). Among these, LBH589 inhibits the proliferation and long-term survival of colorectal cancer cells by DAPK1 activation via induction of Ser308 dephosphorylation and DAPK1 protein levels, inducing DAPK1-dependent autophagy (122). Trichostatin A promotes apoptosis in A549 lung cancer cells and enhances their sensitivity to cisplatin, mediated by upregulating DAPK1 expression and downregulating DAPK1 Ser308 phosphorylation (123). Sodium butyrate induces the expression of DAPK1 in human gastric cancer cells and promotes cell apoptosis by reducing the level of FAK (124). In addition, DAPK1 expression promoted apoptosis by reducing FAK protein level in sodium butyrate treated Raji cells (125). In addition, DNA methyltransferase inhibitors, Vidaza (5-azacytidine) (17) and Decitabine (5-aza-2’-deoxycytidine) (126, 127), which were approved by the FDA, restored DAPK1 expression by DAPK1 promoter hypomethylation in different cancers, indicating an interesting therapeutic option for promoting re-expression of the silenced DAPK1 gene. Sodium selenite triggers autophagy cell death pathway by upregulating and activating DAPK1 in HL60 cells, resulting in cell death (128). Curcumin upregulates the mRNA and protein levels of DAPK1, inhibits STAT3 and NF-κB, activates caspase-3, and induces a G2/M cell cycle arrest and apoptosis in glioblastoma multiforme U251 cells (129). Other small molecules that can also regulate DAPK1 protein levels include lysosomal inhibitors such as E-64d, chloroquine, and leupeptin, as well as the proteasome inhibitor MG132 (43).

### 4.2 Regulation DAPK1 kinase activity

**Figure 7:**
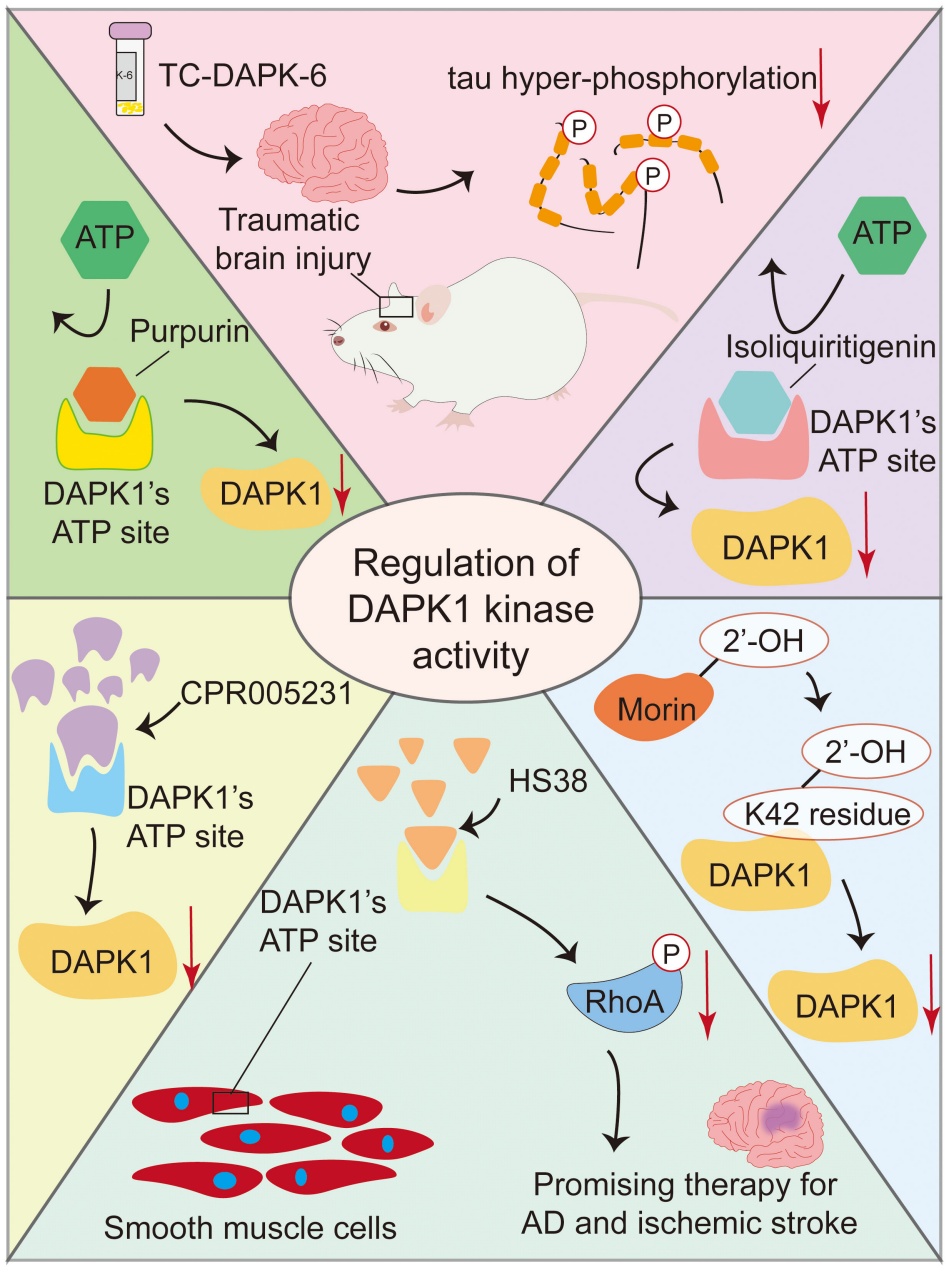
Small molecule inhibitors of DAPK1 kinase activity. This figure summarizes several small molecules that inhibit DAPK1 kinase activity. TC-DAPK6 reduces tau hyper-phosphorylation and anxiety in traumatic brain injury mice. HS38 and CPR005231 are potent inhibitors that bind to DAPK1’s ATP pocket, showing promise for treating AD and ischemic stroke. Morin and Isoliquiritigenin, natural compounds, inhibit DAPK1 via specific binding, with Morin’s halogen derivatives enhancing its effects. Purpurin, a natural 1,2,3-trianthraquinone, also binds to DAPK1’s ATP site. These inhibitors offer therapeutic potential for neurodegenerative and ischemic conditions.

Several small molecules have been identified as inhibitors of DAPK1 kinase activity, with varying potencies and mechanisms of action(**Figure 7**). TC-DAPK6, a DAPK1 kinase activity inhibitor, reduced tau hyper-phosphorylation and anxiety levels in traumatic brain injury mice, suggesting its potential as a therapeutic intervention agent for traumatic brain injury and related neurodegenerative disorders (130). HS38 is a potent inhibitor of DAPK1 with an IC_50_ of 200 nM, which binds to its ATP pockets with Kd values of 300 nM. It shows promise in treating conditions like AD and ischemic stroke by reducing RhoA phosphorylation and related effects in smooth muscle cells, indicating its ability to modulate DAPK1 activity and offer therapeutic benefits (131). CPR005231 is a novel and potent DAPK1 inhibitor that binds to the ATP pocket of DAPK1 with an IC50 value of 247 nM and a Kd of 240 nM, effectively inhibiting its kinase activity, thereby showing promise as a therapeutic candidate for AD and ischemic stroke, with its potency linked to favorable enthalpic changes (132). Morin, a natural flavonoid from the mulberry family Moraceae species, inhibits DAPK1 with an IC_50_ value of 11 µM by binding to its K42 residue via its 2’-OH group, making it a promising lead candidate for drug development for DAPK1-related conditions (83, 133). Isoliquiritigenin is a natural chalcone that acts as an ATP-competitive inhibitor of DAPK1, displaying potential as a therapeutic agent for AD and cerebral ischemia (134). Its halogen derivatives, particularly the chlorine derivative, have been synthesized to enhance inhibitory effects on DAPK1, offering promise for developing new DAPK1 inhibitors. Although its specific role in neurons has not been fully elucidated, Morin is considered a strong candidate for drug development due to its ability to moderately inhibit the catalytic activity of DAPK1. Purpurin, a natural 1,2,3-trianthraquinone isolated from the roots of the plant *Rubia tinctorum*, inhibits DAPK1 with a Kd of 0.37 µM by binding to its ATP site, showing enthalpically favorable binding and serving as a promising lead for DAPK1 inhibitor development (135).

### 4.3 Indirect regulators of DAPK1

**Figure 8:**
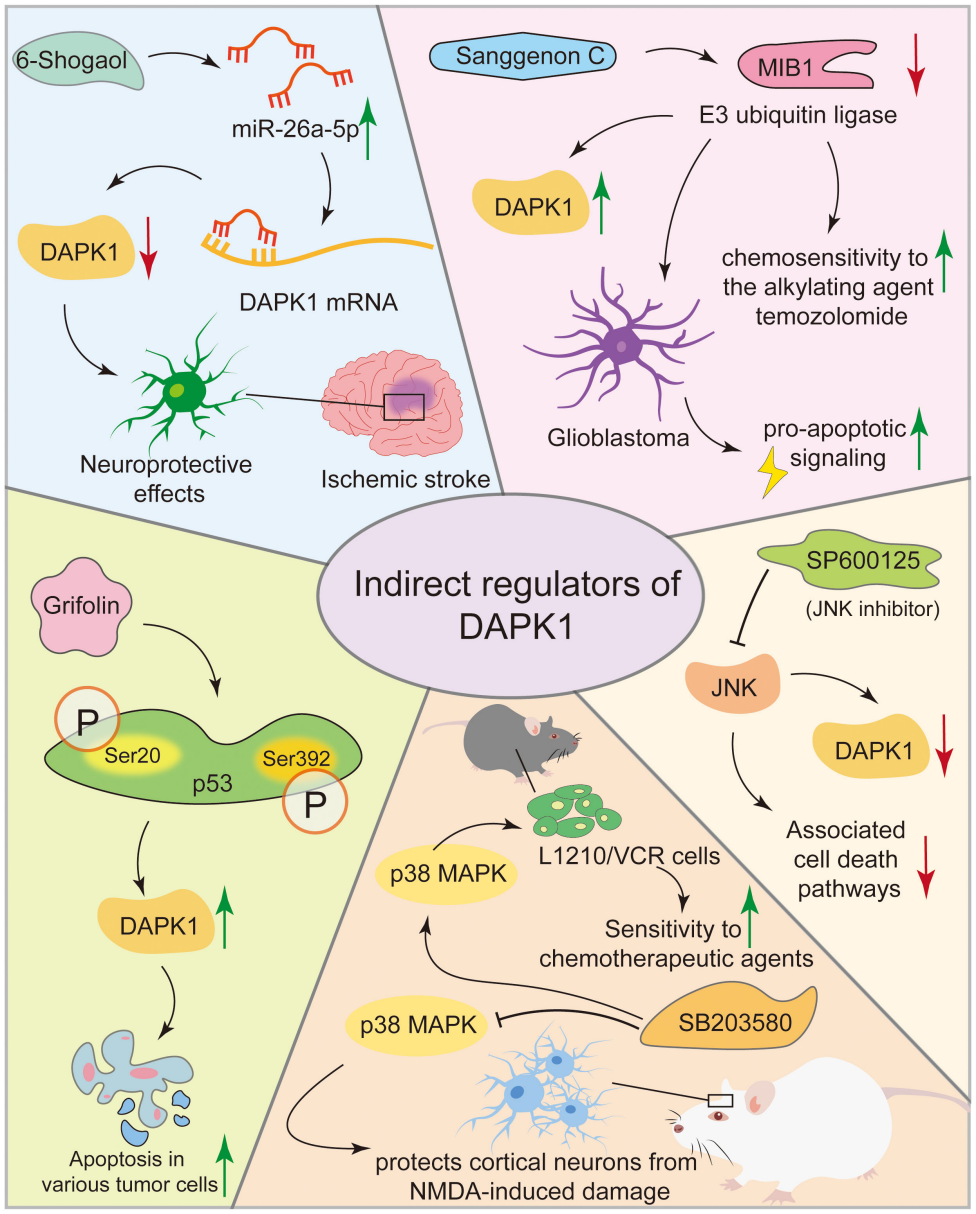
Compounds modulating DAPK1 expression and activity. This figure illustrates various compounds that modulate DAPK1 expression and activity indirectly. In ischemic stroke models, 6-Shogaol reduces neuronal damage by upregulating miR-26a-5p, which inhibits DAPK1 expression. Sanggenon C stabilizes DAPK1 protein in glioblastoma, enhancing chemosensitivity. SP600125, a JNK inhibitor, and SB203580, a p38 MAPK inhibitor, indirectly regulate DAPK1 activity, offering therapeutic benefits in neurodegenerative diseases and cancer. Grifolin induces apoptosis in tumor cells by upregulating DAPK1 via p53 phosphorylation.

In ischemic stroke models, 6-Shogaol indirectly reduced neuronal damage by upregulating miR-26a-5p, which targets DAPK1 mRNA for translational inhibition, thereby suppressing DAPK1 expression (136). 6-Shogaol exerts neuroprotective effects against cerebral ischemia-reperfusion injury by indirectly inhibiting DAPK1 activity through downregulating its expression and modulating phosphorylation, thereby alleviating calcium overload and excessive neuronal autophagy (137). Sanggenon C paradoxically stabilizes DAPK1 protein by inhibiting the E3 ubiquitin ligase MIB1, leading to DAPK1 accumulation and pro-apoptotic signaling in glioblastoma, enhancing chemosensitivity to the alkylating agent temozolomide (38). SP600125, a selective JNK inhibitor, indirectly regulates DAPK1 activity by inhibiting the JNK pathway, thereby offering potential therapeutic benefits in neurodegenerative diseases and cancer by reducing DAPK1 activation and associated cell death pathways (138, 139). In neurodegenerative diseases like AD and ischemic stroke where overactive DAPK1 contributes to neuronal cell death, SB203580 protected primary rat cortical neurons from NMDA-induced damage by inhibiting the p38 MAPK pathway, which in turn reduced DAPK1 activation and mitigated neuronal toxicity (140). In cancer, SB203580, a specific inhibitor of p38α and p38β which suppresses downstream activation of MAPKAP kinase-2 and heat shock protein 27, reduced vincristine resistance in mouse leukemia L1210/VCR cells, presumably by modulating the p38 MAPK pathway, thereby activating DAPK1 via its dephosphorylation at Ser308, thus increasing sensitivity to chemotherapeutic agents (141, 142). Grifolin, a compound isolated from the fresh fruiting bodies of the mushroom *albatrellus confluens*, induced apoptosis in various tumor cells by upregulating DAPK1 expression and activity by enhancing p53 phosphorylation at Ser 20 and Ser392. This increased the transcriptional activity of p53 and its binding to the DAPK1 gene promoter, resulted in elevated DAPK1 mRNA and protein levels in a dose-dependent manner (143, 144). However, none of these compounds can specifically target DAPK1 expression, complicating the interpretation of the role of DAPK’ in the cellular effects elicited by these above agents (**Figure 8**). Consequently, despite the availability of numerous compounds capable of activating DAPK1, no specific clinical candidate has been identified that exclusively activates DAPK1. This does not imply that these compounds or their analogs in clinical trials, lack utility in DAPK1 activation. For instance, if a nonspecific compound such as a methylation inhibitor demonstrates low toxicity in patients and its therapeutic efficacy is contingent upon DAPK1 activation, it may be considered a viable DAPK1 activator for possible clinical applications. Further research is required to elucidate the relationship between the clinical outcomes of these compounds and DAPK1 activation.

## 5 DAPK1 distribution

Determination of the distribution of DAPK1 in different tissues and cell types is crucial for elucidating its functional roles in health and disease.

### 5.1 Tissue distribution

DAPK1 mRNA is extensively expressed in the developing and adult central nervous system (CNS) of rats, with its presence noted in both proliferative and postmitotic cells within the cerebral cortex, hippocampus, and cerebellum from embryonic day 13 onward (149, 150). Postnatally, there is a significant reduction in DAPK1 mRNA levels in the brain; however, it remains elevated in specific neuronal populations, including those in the olfactory bulb, hippocampus, as well as cerebellar Purkinje and granule cells (149). This temporal and spatial regulation of DAPK1 expression implies a potential role in developmental neuronal cell death. Although no CNS abnormalities have been reported in DAPK1 knockout mice, the DAPK1 deletion conferred neuroprotective effects against various toxic insults (99, 151, 152). In skeletal and cardiac muscle, DAPK1 is implicated in muscle cell differentiation and survival, with its expression in these tissues being linked to stress responses, such as ischemia, where DAPK1 may facilitate cell death in response to oxidative stress (28, 153).

**Figure 9:**
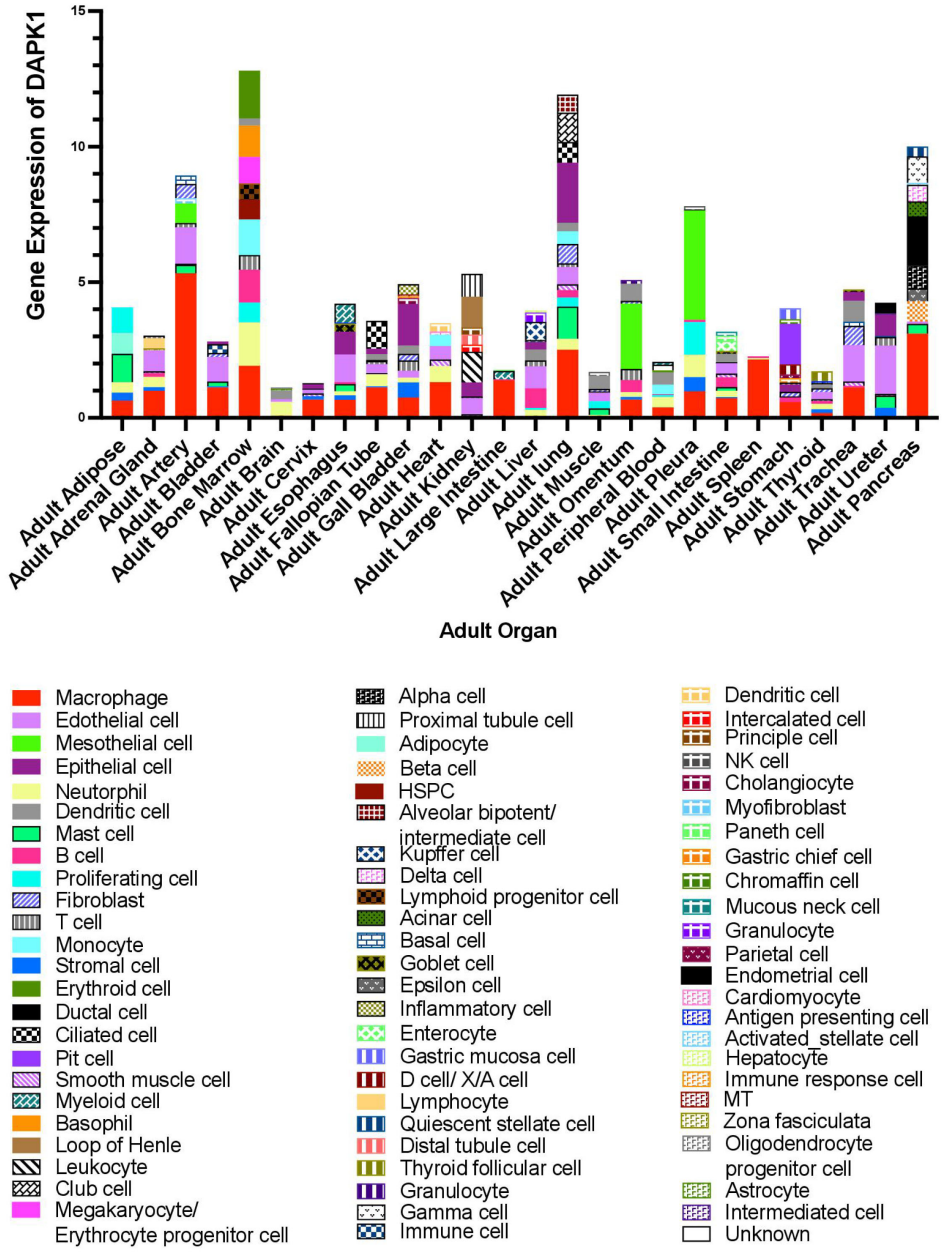
Online database analysis of DAPK1 gene expression in various organs using single-cell data from the HCL Landscape webpage (https://bis.zju.edu.cn/HCL/landscape.html).

DAPK1 is also expressed in various immune cells, including macrophages, T cells, and B cells (71, 154, 155). In these cells, DAPK1 modulates inflammatory responses and apoptosis, influencing immune system function (154, 156). DAPK1 expression is upregulated in response to cytokines and other inflammatory stimuli, highlighting its role in immune responses. Additionally, through single-cell analysis via the webpage HCL Landscape https://bis.zju.edu.cn/HCL/landscape.html, we found that DAPK1 is mainly expressed in macrophages in the arteries, heart, large intestine, spleen, and cervix of adults. In the liver, DAPK1 is prominently expressed in Kupffer cells, which are specialized macrophages residing in the liver sinusoids. Kupffer cells play a critical role in maintaining liver homeostasis and immune surveillance (157). The localization of DAPK1 in these cells indicates its potential involvement in liver-specific immune responses and the regulation of Kupffer cell functions, such as the clearance of pathogens and damaged cells (**Figure 9**).

### 5.2 Subcellular localization of DAPK1

**Figure 10:**
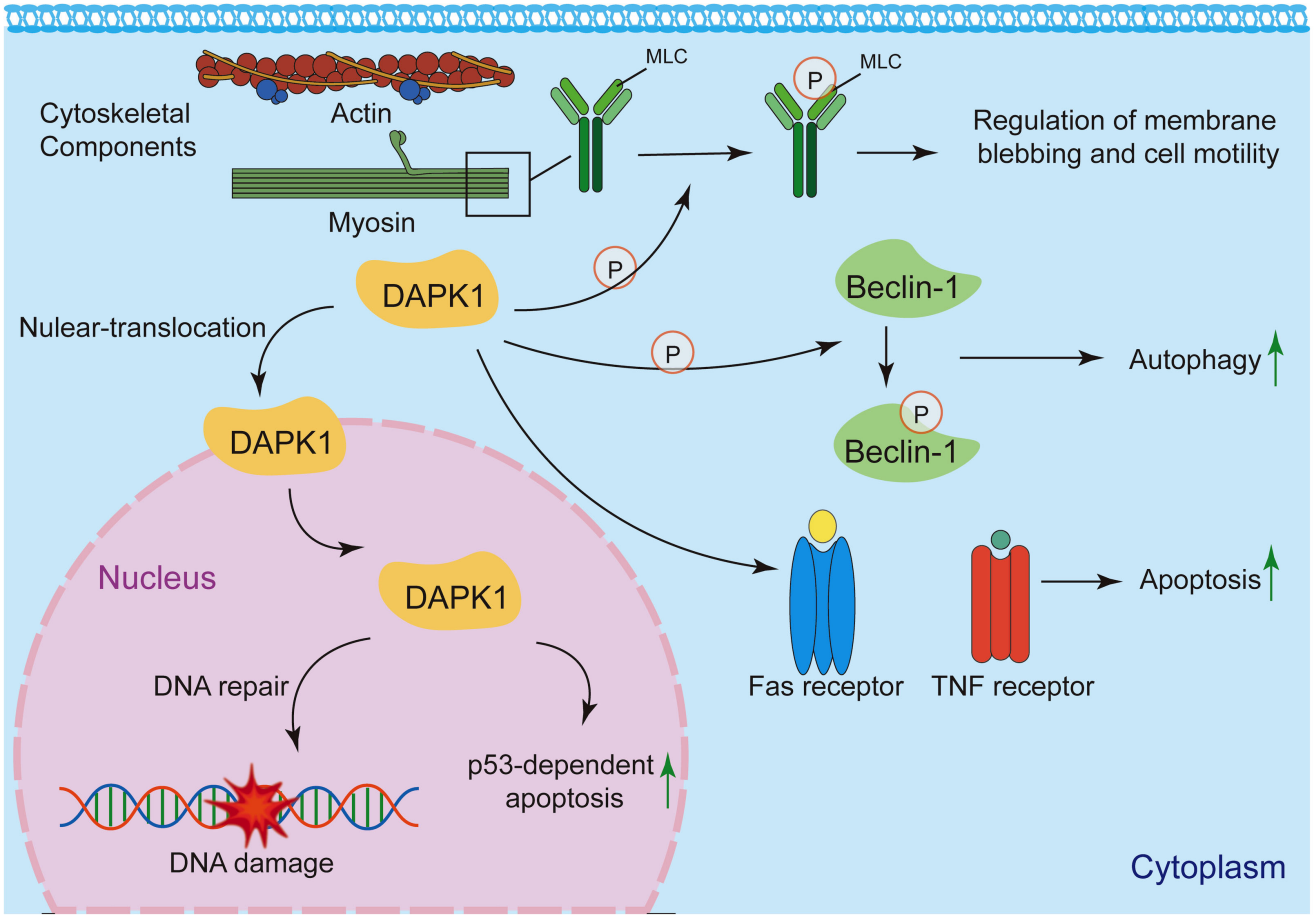
Subcellular localization and functions of DAPK1. Under basal conditions, DAPK1 is mainly located in the cytoplasm, where it regulates cytoskeletal reorganization, autophagy, and apoptosis signaling. It interacts with actin and myosin to control membrane blebbing and cell motility, and phosphorylates myosin light chain (MLC) to induce actomyosin contractility. DAPK1 also promotes autophagy by phosphorylating Beclin-1 and participates in death receptor-mediated apoptosis. Under specific conditions like DNA damage, DAPK1 can translocate to the nucleus and contributes, to p53-dependent apoptosis.

Under basal conditions, DAPK1 is predominantly localized in the cytoplasm (158) (**Figure 10**). This localization is critical for its involvement in cytoskeletal reorganization, autophagy induction, and extrinsic apoptosis signaling (18, 21, 62, 66, 67). DAPK1 interacts with cytoskeletal components, such as actin and myosin, to regulate membrane blebbing and cell motility (18, 72, 158, 159). Its cytoplasmic localization facilitates the phosphorylation of myosin light chain (MLC), leading to actomyosin contractility and membrane blebbing during apoptosis (18). DAPK1 promotes autophagy by phosphorylating Beclin-1, a key autophagy regulator, in the cytoplasm (67). This process is essential for cellular homeostasis and stress responses. DAPK1 is involved in death receptor-mediated apoptosis by interacting with cytoplasmic signaling complexes, such as those involving Fas and TNF receptors (56). Under specific conditions, such as DNA damage, DAPK1 can translocate to the nucleus, where it may participate in nuclear-specific functions (62, 158). DAPK1 has been implicated in p53-dependent apoptosis following DNA damage (62). Its nuclear translocation may facilitate interactions with nuclear proteins involved in DNA repair or apoptosis.

The subcellular localization of DAPK1 is a key determinant of its functional diversity. While primarily localized in the cytoplasm, DAPK1 can translocate to the nucleus under specific conditions, where it may engage in distinct signaling pathways. Further research is needed to elucidate the precise mechanisms governing DAPK1 localization and its functional consequences in health and disease.

## 6 Future perspectives

### 6.1 Differential expression of DAPK1 in various organs and cell types

Over the past decade, it has become increasingly evident that a single gene may exhibit entirely opposing functions in different cell types (160–163). While some studies have explored the role of DAPK1 in various cell types including immune cells, endothelial cells, and epithelial cells, our understanding of DAPK1 expression and function across diverse cellular contexts—especially within complex tissues like cancer—remains limited. The advent of modern technologies, such as single-cell sequencing, has increasingly enabled us to investigate gene distribution at the cellular level (164, 165). By leveraging online databases, we have delineated in the current review the intricate distribution of DAPK1 across various cell types in different organs (**Figure 9**). This highlights the necessity of considering cellular context when examining the functional roles of DAPK1. This is particularly relevant in the field of cancer, where a significant proportion of DAPK1-expressing cells are tumor-associated macrophages (**Figure 9**) (101, 166). The differential expression of DAPK1 in various cell types may explain some of the conflicting findings in cancer research, especially those involving the use of clinical specimens for prognostic analysis. The cell types present in these samples can vary dramatically depending on the sampling site, thereby influencing the results. Moreover, as an important tumor suppressor, most studies on DAPK1 in cancer have focused on epithelial cells. Our observations suggest that DAPK1 may also play a crucial role in the tumor microenvironment (TME), warranting further investigation. This is supported by recent findings that DAPK1 influences the activation and trafficking of CD8+ T cells within the TME, enhancing their antitumor activity and potentially modulating the immune response against tumors (167).

### 6.2 Differential functions of DAPK1 signaling pathway in various cell types

The complexity of gene signaling pathways has long been recognized, with genes like p53 having over 300 downstream targets and more than 100 upstream regulators (168, 169). Nowadays, our focus may need to shift from discovering additional gene-gene interactions to the identification of more specific contexts in which given signaling pathways function. This is equally applicable to DAPK1. As previously mentioned, we can now precisely determine the distribution of DAPK1 in different cell types. It would be intriguing to compare the impact of the same DAPK1 pathway across distinct cell types. For instance, is the DAPK1-ARF-p53 pathway functional in macrophages, and what are the consequences of its activation? These questions warrant further in-depth exploration.

### 6.3 Common DAPK1 regulatory mechanisms underlying disease development

Despite DAPK1’s involvement in numerous diseases and the diversity of its molecular mechanisms, it is plausible that many diseases share common regulatory mechanisms, either directly or indirectly. For example, several studies have reported that DAPK1 phosphorylates myosin light chain at Ser19, the very same site targeted by MLCK (11, 170, 171). This phosphorylation affects cell movement and contraction in cancer and is also a critical site in hypertension, where overactivation of MLCK-dependent phosphorylation of this Ser19 is a key pathological factor (172, 173). Furthermore, the high expression of DAPK1 in macrophages suggests that it may impact the M1/M2 polarization of M0 macrophages, which are actively involved in the development of many diseases. Therefore, it would be valuable to resolve the potential roles of DAPK1 in various diseases, as well as the upstream stimuli and downstream pathways, based on existing regulatory pathways before conducting further validation.

### 6.4 Potential DAPK1 related diseases

Based on current research, several potential disease areas appear to be promising candidates for future investigation. First, as previously discussed, DAPK1 may play a crucial role in hypertension (18, 93, 173). This is because its downstream target, MLC, is integral to the renin-angiotensin-aldosterone system (RAAS)-induced hypertension pathway (174). Second, studies have reported that ER stress-induced DAPK1-dependent xenophagy, can counteract mitochondrial stress-induced epithelial barrier dysfunction (175, 176). This mechanism helps suppress inflammation and mitigates dextran sodium sulfate (DSS)-induced colitis, a classic model of inflammatory bowel disease (IBD). These findings suggest that DAPK1 may be actively involved in regulating IBD. Third, it has been reported that pegylated IFN-α can suppress hepatitis C virus (HCV) by promoting the DAPK1-mTOR pathway (177). Additionally, DAPK1 has been found to be involved in the regulation of hepatitis B virus (HBV) and Epstein-Barr virus (EBV) infections (178–180). These studies indicate that the DAPK1 pathway may play important roles in regulating DNA virus infections, thus warranting further investigation.

## 7 Conclusions

Although DAPK1 was initially identified as a significant tumor suppressor, the expanding network of its upstream and downstream pathways has potentially extended its roles to numerous diseases. The fact that DAPK1 knockout mice can develop normally, similar to p53 knockout mice, suggests that DAPK1 may function as a stress sensor, remaining dormant under healthy conditions, much like p53. Moreover, given the complex and dual roles of DAPK1 in both promoting and suppressing inflammation—mirroring p53’s role as the “guardian of the genome”—it is reasonable to propose that DAPK1 may serve as one of the “guardians of inflammation.” Further research across diverse disease models will be essential to validate this hypothesis and elucidate the multifaceted functions of DAPK1.
